# High- and Low-Fat Dairy Consumption and Long-Term Risk of Dementia: Evidence From a 25-Year Prospective Cohort Study

**DOI:** 10.1212/WNL.0000000000214343

**Published:** 2025-12-17

**Authors:** Yufeng Du, Yan Borné, Jessica Samuelsson, Isabelle Glans, Xiaobin Hu, Katarina Nägga, Sebastian Palmqvist, Oskar Hansson, Emily Sonestedt

**Affiliations:** 1 Department of Epidemiology and Statistics, School of Public Health, Lanzhou University, Gansu, China;; 2 Nutritional Epidemiology, Department of Clinical Sciences Malmö, Lund University, Sweden;; 3 Neuropsychiatric Epidemiology Unit, Department of Psychiatry and Neurochemistry, Institute of Neuroscience and Physiology, Sahlgrenska Academy, Centre for Ageing and Health at the University of Gothenburg, Mölndal, Sweden;; 4 Clinical Memory Research Unit, Department of Clinical Sciences Malmö, Lund University, Sweden;; 5 Memory Clinic, Skåne University Hospital, Malmö, Sweden;; 6 Department of Geriatrics and Palliative Medicine, and Department of Health, Medicine and Caring Sciences, Linköping University, Sweden; and; 7 Department of Food and Meal Science and the Research Environment MEAL, Faculty of Natural Science, Kristianstad University, Sweden.

## Abstract

**Background and Objectives:**

The association between dairy intake and dementia risk remains uncertain, especially for dairy products with varying fat contents. The aim of this study was to investigate the association between high-fat and low-fat dairy intake and dementia risk.

**Methods:**

This study used data from a prospective cohort in Sweden, the Malmö Diet and Cancer cohort, which consisted of community-based participants who underwent dietary assessment at baseline (1991–1996). Dietary intake was evaluated using a comprehensive diet history method that combined a 7-day food diary, a food frequency questionnaire, and a dietary interview. Dementia cases were identified through the Swedish National Patient Register until December 31, 2020, and cases diagnosed until 2014 were further validated. The primary outcome of the study was all-cause dementia, and the secondary outcomes were Alzheimer disease (AD) and vascular dementia (VaD). Cox proportional hazard regression models were used to estimate hazard ratio (HR) and 95% CI.

**Results:**

This study included 27,670 participants (mean baseline age 58.1 years, SD 7.6; 61% female). During a median of 25 years of follow-up, 3,208 incident dementia cases were recorded. Consumption of ≥50 g/d of high-fat cheese (>20% fat) was associated with a reduced risk of all-cause dementia (HR 0.87; 95% CI, 0.78–0.97) and VaD (HR 0.71, 95% CI 0.52–0.96) compared with lower intake (<15 g/d). An inverse association between high-fat cheese and AD was found among *APOE* ε4 noncarriers (HR 0.87, 95% CI 0.76–0.99, *p*-interaction = 0.014). Compared with no consumption, individuals consuming ≥20 g/d of high-fat cream (>30% fat) had a 16% lower risk of all-cause dementia (HR 0.84, 95% CI 0.72–0.98). High-fat cream consumption was inversely associated with the risk of AD and VaD. Consumption of low-fat cheese, low-fat cream, milk (high-fat and low-fat), fermented milk (high-fat and low-fat), and butter showed no association with all-cause dementia.

**Discussion:**

Higher intake of high-fat cheese and high-fat cream was associated with a lower risk of all-cause dementia, whereas low-fat cheese, low-fat cream, and other dairy products showed no significant association. *APOE* ε4 status modified the association between high-fat cheese and AD. Our study's observational design limits causal inference.

## Introduction

According to the 2019 Global Burden of Disease Study, dementia cases are projected to rise sharply, from 57 million in 2019 to 153 million by 2050.^1^ Because effective treatments for dementia are lacking, identifying preventive approaches through risk factor modification is essential to reduce its burden. Diet is considered one of the modifiable risk factors.^2^ A dietary pattern aimed at reducing the risk of cognitive decline and dementia (i.e., the Mediterranean Dietary Approaches to Stop Hypertension Intervention for Neurodegenerative Delay [MIND] diet) has shown inconsistent associations with dementia risk, with some studies reporting no association^3,4^ while others suggest a protective association.^5-8^ In a recent randomized controlled trial (RCT), the MIND diet did not significantly improve cognitive function or brain imaging outcomes compared with a control diet.^9^

Dairy products, an important component of traditional Western diets, have been extensively studied for their associations with various health outcomes.^10,11^ However, its association with dementia is still debated. Total dairy intake has shown a protective association with dementia in Asian populations, but not in European populations.^12^ Dairy products vary in processing methods, food matrix, and fat and nutrient content, which may influence their health effects.^13^ Therefore, it is important to examine specific dairy products separately in relation to dementia risk. Among dairy products, cheese seems to have the strongest potential to protect against cognitive decline.^14^ The association between dementia and cream intake remains underexplored while limited evidence on milk and yogurt suggests null associations.^12,14-17^ In the MIND diet, cheese was categorized as an unhealthy food to limit.^18^ However, a Finnish cohort study of 2,497 men showed a 28% lower dementia risk with higher cheese intake.^15^ A UK Biobank cohort study with 11 years of follow-up found that consuming cheese once a week was linked to a lower dementia risk compared with no consumption.^19^ By contrast, a cohort study from Japan (n = 11,637) with 5 years of follow-up found no association.^16^ Cross-sectional studies from Japan, the Netherlands, and the United Kingdom have linked higher cheese intake to better cognitive function in older adults.^4,20-22^ *APOE* ε4, the leading genome-wide genetic risk variant for sporadic Alzheimer disease (AD), may modify the association between diet and dementia/AD risk.^23-27^ However, the study examining dairy products found no significant interactions with *APOE* ε4 status.^15^

Although previous studies provide insights, they have limitations such as small sample sizes, cross-sectional designs, and a lack of differentiation between dementia subtypes. In addition, relatively short follow-up periods may introduce reverse causation bias, considering the long preclinical phase of dementia (up to 15–20 years).^28^ While high-fat and low-fat dairy products have been differentially associated with the risk of all-cause, cardiovascular disease (CVD), and cancer mortality,^29^ it remains unknown whether they are differentially linked to dementia risk.

The aim of this study was to examine the associations between intake of high-fat and low-fat dairy and the risk of all-cause dementia, AD, and vascular dementia (VaD) using data from the Malmö Diet and Cancer (MDC) cohort. We also examined whether the *APOE* ε4 status modified these associations.

## Methods

### Standard Protocol Approvals, Registrations, and Participant Consents

The MDC study was approved by the Ethical Committee at the Medical Faculty at Lund University (approval number: LU 51/90). All participants provided written informed consent.

### Study Population

The MDC is a prospective cohort in which participants aged 45–73 years were recruited in Malmö, Sweden, between 1991 and 1996. A total of 30,446 of the 74,318 invited individuals attended the baseline examination. Individuals unable to complete the baseline questionnaire because of language barriers or cognitive impairment were excluded. Participants received a self-administered questionnaire covering lifestyle factors and sociodemographic characteristics, along with a food diary and a food frequency questionnaire (FFQ), at their first visit. Trained personnel performed anthropometric measurements and blood sample collection. Participants returned questionnaires and underwent a dietary interview during their second visit, approximately 2 weeks after the first. The MDC design has been described in detail elsewhere.^30,31^

Of the 28,098 participants completing the baseline examination, 428 were excluded because of missing covariate data, leaving 27,670 participants for analysis. The analysis of *APOE* ε4 and dairy interaction was restricted to 26,661 participants with available genetic data (18,606 noncarriers; 8,055 carriers). Details are presented in a flowchart (eFigure 1).

### Exposure Assessment

Dietary intake was evaluated using a modified diet history approach that integrated a 7-day food diary, a semiquantitative FFQ containing 168 items, and a dietary interview lasting 45–60 minutes. The method was validated by comparison with 18-day weighed food records, yielding energy-adjusted correlation coefficients (validity/reproducibility) for milk (female 0.84/0.70, male 0.83/0.82), cheese (female 0.59/0.71, male 0.47/0.71), and cream (female 0.52/0.42, male 0.47/0.48).^32,33^ Participants recorded their cooked meals (mainly lunch and dinner), medications, dietary supplements, and cold beverages for 7 consecutive days in the food diary. The FFQ captured information on regularly consumed foods that were not covered in the food diary (i.e., breakfast, hot beverages, and snacks), over the previous year. Dairy used in cooked meals and milk (as a drink) from 3 prespecified types with various fat contents were recorded in the food diary while the intake of other dairy products not covered in the 7-day food dairy such as milk and cream added to coffee, milk in tea, milk in porridge and breakfast cereals, cheese on bread, other cheeses (e.g., cheese plates), yogurt, and other fermented milks (e.g., sour milk) was mainly assessed through the FFQ.^34^ The dietary interview aimed to quantify foods recorded in the food diary, evaluate the overlap between the food diary and FFQ, and collect details on food preparation. The collected food data were aggregated to determine daily intake of each food item (grams per day), and energy and nutrient intake was assessed using the Swedish Food Database (PC-KOST2-93) from the Swedish National Food Administration.

The fat thresholds used to differentiate between low-fat and high-fat dairy products were 2.5% for milk and fermented milk, 20% for cheese, and 30% for cream; these thresholds were based on the prespecified fat contents in the original items of the FFQ. The threshold for high-fat cheese and milk in our study was consistent with previous studies.^35^ We categorized intake of each dairy products into 4 categories based on the distribution.

### Outcome Ascertainment

Dementia cases were identified using the Swedish National Patient Register (NPR), which includes both the Swedish Inpatient Register and the hospital-based outpatient register. Diagnoses were based on codes from the ninth and tenth versions of the International Classification of Diseases (ICD), including the following: AD dementia (ICD-10 and ICD-9 codes F00, G30, 331A/331.0), VaD (F01, 290E/290.4), Parkinson disease dementia (F020, G310, 331B/331.1), and unspecified dementia (F03, 290, 294B/294.1, 331C/331.2). Trained physicians at the Memory Clinic, Skåne University Hospital, have reviewed and validated all dementia diagnoses until December 31, 2014, based on symptoms, cognitive test results, brain imaging, and CSF levels of amyloid-β42 and phosphorylated-tau (when available), following Diagnostic and Statistical Manual of Mental Disorders, Fifth Edition criteria. In uncertain cases, 2 specialists in Neurology and Geriatrics, each with over 10 years of dementia experience, were consulted.^36,37^ The validation was not continued for cases diagnosed after 2014, mainly because of resource limitations. Of the NPR-diagnosed all-cause dementia cases, 96% were confirmed through the validation process.^37^ Consequently, all-cause dementia registered in the NPR is considered accurate. However, this is not the case for the NPR-based specific dementia diagnosis (e.g., AD or VaD), of which 40% were revised during the validation process.^37^

The analysis of all-cause dementia included cases diagnosed before December 31, 2014 (all validated cases), and cases diagnosed before December 31, 2020 (all validated cases through 2014 plus unvalidated cases from 2015 to 2020). For dementia subtypes, only validated cases diagnosed before 31 December 2014 were included.

Participants were followed up from the baseline examination until the dementia diagnosis, death, emigration, or December 31, 2020 (or December 31, 2014), whichever occurred first. Emigration resulted in 0.75% of participants being lost to follow-up.

### Assessment of Covariates

Age and sex were obtained from the Swedish registry using the personal identification number. Data on marital status (married or others), living alone (yes or no), educational level (elementary, primary and secondary, upper secondary, further education without a degree, and university degree), and smoking status (current, former, or never) were collected through a self-administered questionnaire. Alcohol consumption was classified into 6 categories: zero-consumers and sex-specific quintiles for drinkers. Leisure-time physical activity, based on 17 activities and measured in metabolic equivalent task (MET) hours per week, was grouped into 5 categories (<7.5, 7.5–15, 15–25, 25–50, and >50 MET hour/week). The heredity score for CVD was determined by self-reported family history (parents or siblings). The diet quality index (range 0–5) was based on 5 components: fiber, added sugar, fruits and vegetables, fish, and red and processed meat.^38^ The dietary assessment method (old or new) was included as a covariate because the interview duration was reduced from 60 to 45 minutes after September 1994. The date of dietary data collection was classified into seasons.

Body mass index (BMI) was calculated from measured weight and height and categorized into 4 groups (<18.5, 18.5–24.9, 25–29.9, and ≥30 kg/m^2^). Hypertension was defined as systolic blood pressure ≥140 mm Hg and/or diastolic blood pressure ≥90 mm Hg, or the use of antihypertensive medication. Diabetes was identified through self-reported diagnosis, medication usage, and registry records. The intake of lipid-lowering drugs was self-reported. Baseline blood lipoproteins were only available in 4,549 participants and, therefore, not included as covariates. The *APOE* genotype was determined based on 2 single-nucleotide variants, *rs429358* and *rs7412*, which define the ε2, ε3, and ε4 alleles. Participants carrying at least 1 ε4 allele were classified as *APOE* ε4 carriers. During the 5-year follow-up examination (1997–2001), participants were asked, “Have you substantially changed your dietary habits since you participated in the MDC study for the first time?” to assess changes in diet.

### Statistical Analyses

Baseline characteristics are reported as mean ± SD for continuous variables and as n (%) for categorical variables. We used Cox proportional hazard regression models to estimate hazard ratio (HR) and 95% CI. *p* for trend was estimated by entering the median value of each intake group as a continuous variable in Cox models. Covariates were progressively adjusted in 2 models. Model 1 was adjusted for age, sex, season, dietary assessment method, and total energy intake. Model 2 was further adjusted for marital status, living alone, educational level, leisure-time physical activity, smoking status, alcohol consumption, heredity score for CVD, diet quality index, BMI, hypertension, and mutual adjustment for dairy products (i.e., high-fat and low-fat cheese, cream, milk, fermented milk, and butter). The proportional hazard assumption was evaluated with the Schoenfeld test, and the results indicated no violation. Potential nonlinear associations between dairy intake and dementia were explored using the restricted cubic splines model. We optimized the number of knots for the splines by minimizing the Akaike Information Criterion, based on 4 models using 3 to 6 knots. Three knots were optimal for most models, except for all-cause dementia (2014) and low-fat milk (4 knots).

We performed stratified analyses by sex, age, educational level, diet quality index, and *APOE* ε4 status (noncarriers/carriers) and examined potential interactions using the Wald test on cross-product terms (dairy intake × stratification variables).

We performed a series of sensitivity analyses. First, to minimize the chance of potential reverse causation, we repeated model 2 after the following exclusions: excluding dementia cases occurring within the first 10 years of follow-up (n = 345) or excluding prevalent CVD, cancer, or diabetes cases at baseline (n = 3,518). Second, model 2 was further adjusted for cancer, CVD, diabetes, and lipid-lowering medication. Last, we restricted our analysis to those who reported no substantial change in their diet during the 5-year follow-up examination (n = 18,296).

Substitution analyses were conducted as post hoc analyses to examine the risk changes when replacing 20 g/d of high-fat cheese and cream with an equivalent amount of other foods with different fat contents, that is, white meat, high-fat red meat (>10% fat), low-fat red meat (≤10% fat), processed meat, desserts and snacks (biscuits, cakes/pastries, ice cream, sorbet, crackers, wafers, wheat rusks, and coffee bread), high-fat margarine and mayonnaise (>40% fat), and low-fat margarine and mayonnaise (≤40% fat). The HRs and 95% CI for the substitution association were estimated by the difference in coefficients, variance, and covariance.^39^

We used R version 4.2.1 (R Foundation, Vienna, Austria) and SAS software version 9.4 (SAS Institute, Cary, NC) for statistical analyses. All tests were 2-sided and significant at 0.05.

### Data Availability

The data used in this study are sourced from the Malmö Population-Based Cohorts Joint Database and were accessed under license. Owing to licensing restrictions, the data are not publicly accessible. However, they can be made available by the corresponding author on reasonable request, with permission from the MDC steering committees.

## Results

### Participant Characteristics

This study included 27,670 participants (61% female), with an average age of 58.1 years at baseline (range 45–73 years). Baseline characteristics of participants are presented in Tables 1 and 2. Compared with those with the lowest intake of high-fat cheese or high-fat cream (Table 1), those in the highest intake group were more likely to have lower BMI and higher education levels. They also had a lower prevalence of diabetes, hypertension, CVD, and stroke and were less likely to use lipid-lowering medication. Participants who consumed more low-fat cheese were more likely to be female, past or never smokers, and physically active; have higher diet quality index; and have a higher prevalence of diabetes or CVD. Individuals who consumed more low-fat milk or low-fat fermented milk were more likely to use lipid-lowering medication and have chronic conditions compared with nonconsumers, whereas those with higher intakes of high-fat milk, high-fat fermented milk, or butter showed the opposite trend (Table 2). Individuals with higher intakes of high-fat milk or butter were more likely to be male, current smokers, and unmarried; have a lower BMI; and have poorer diet quality.

**Table 1:** Baseline Characteristics by Categories of Cheese and Cream Consumption^a^

Characteristics	Total	High-fat cheese		Low-fat cheese		High-fat cream		Low-fat cream	
		<15 g/d	≥50 g/d	0 g/d	≥30 g/d	0 g/d	≥20 g/d	0 g/d	≥20 g/d
Participants, n	27,670	7,340	7,001	16,318	3,051	7,149	2,121	5,952	3,201
Age, y	58.1 ± 7.6	59.4 ± 7.5	56.3 ± 7.3	58.5 ± 7.7	57.6 ± 7.1	58.6 ± 7.0	58.0 ± 7.6	57.5 ± 7.7	59.6 ± 7.3
Female	16,787 (60.7)	4,526 (61.7)	4,005 (57.2)	9,082 (55.7)	2,063 (67.6)	3,827 (53.5)	1,168 (55.1)	3,542 (59.5)	1,792 (56.0)
BMI, kg/m^2^	25.7 ± 4.0	26.2 ± 4.1	25.3 ± 3.9	25.6 ± 3.9	26.0 ± 4.1	26.2 ± 4.1	24.9 ± 3.4	25.8 ± 4.1	25.5 ± 3.8
Married	18,073 (65.3)	4,762 (64.9)	4,363 (62.3)	10,585 (64.9)	1,954 (64.0)	4,574 (64.0)	1,440 (67.9)	3,536 (59.4)	2,165 (67.6)
Living alone	6,734 (24.3)	1,937 (26.4)	1,754 (25.1)	4,005 (24.5)	804 (26.4)	1,897 (26.5)	465 (21.9)	1,723 (29.0)	714 (22.3)
University degree	3,949 (14.3)	718 (9.8)	1,407 (20.1)	1,997 (12.2)	548 (18.0)	655 (9.2)	417 (19.7)	981 (16.5)	334 (10.4)
Smoking status									
Current	7,798 (28.2)	2,012 (27.4)	2,103 (30.0)	5,044 (30.9)	751 (24.6)	2,257 (31.6)	563 (26.5)	1,890 (31.8)	928 (29.0)
Past	9,374 (33.9)	2,461 (33.5)	2,454 (35.1)	5,425 (33.3)	1,139 (37.3)	2,384 (33.4)	744 (35.1)	1,967 (33.1)	1,056 (33.0)
Never	10,498 (37.9)	2,867 (39.1)	2,444 (34.9)	5,849 (35.8)	1,161 (38.1)	2,508 (35.1)	814 (38.4)	2,095 (35.2)	1,217 (38.0)
Zero-consumers of alcohol	1,737 (6.3)	750 (10.2)	299 (4.3)	1,001 (6.1)	254 (8.3)	664 (9.3)	67 (3.2)	548 (9.2)	198 (6.2)
High leisure-time physical activity (>50 MET hours/week)	4,454 (16.1)	1,217 (16.6)	1,137 (16.2)	2,558 (15.7)	536 (17.6)	1,107 (15.5)	375 (17.7)	943 (15.8)	556 (17.4)
Heredity score for CVD (>0)	14,728 (53.2)	4,026 (54.9)	3,587 (51.2)	8,564 (52.5)	1,705 (55.9)	3,726 (52.1)	1,148 (54.1)	3,069 (51.6)	1,717 (53.6)
Chronic conditions at baseline									
Diabetes	1,209 (4.4)	503 (6.9)	217 (3.1)	558 (3.4)	251 (8.2)	406 (5.7)	57 (2.7)	298 (5.0)	136 (4.3)
Hypertension	17,028 (61.6)	4,875 (66.5)	3,905 (55.8)	10,188 (62.5)	1,836 (60.2)	4,701 (65.8)	1,199 (56.6)	3,530 (59.3)	2,068 (64.7)
CVD	829 (3.0)	350 (4.8)	120 (1.7)	477 (2.9)	107 (3.5)	262 (3.7)	37 (1.7)	186 (3.1)	87 (2.7)
Stroke	284 (1.0)	100 (1.4)	49 (0.7)	180 (1.1)	30 (1.0)	80 (1.1)	15 (0.7)	68 (1.1)	32 (1.0)
Cancer	1,713 (6.2)	509 (6.9)	369 (5.3)	1,001 (6.1)	181 (5.9)	407 (5.7)	126 (5.9)	321 (5.4)	222 (6.9)
Lipid-lowering medication	868 (3.1)	439 (6.0)	100 (1.4)	402 (2.5)	138 (4.5)	279 (3.9)	34 (1.6)	174 (2.9)	91 (2.8)
Diet quality index	1.9 ± 1.3	2.1 ± 1.3	1.9 ± 1.2	1.7 ± 1.2	2.5 ± 1.3	1.9 ± 1.3	1.9 ± 1.2	2.0 ± 1.3	1.9 ± 1.2
Total energy intake, kcal/d	2,275.8 ± 653.2	2,038.0 ± 593.2	2,619.6 ± 700.7	2,318.2 ± 672.1	2,246.3 ± 620.9	2,223.0 ± 676.7	2,599.0 ± 671.0	2,158.3 ± 652.6	2,486.0 ± 678.4
All-cause dementia (2014)	1,920 (6.9)	609 (8.3)	368 (5.3)	1,155 (7.1)	212 (7.0)	549 (7.7)	125 (5.9)	370 (6.2)	284 (8.9)
All-cause dementia (2020)	3,208 (11.6)	968 (13.2)	665 (9.5)	1,885 (11.6)	363 (11.9)	892 (12.5)	223 (10.5)	660 (11.1)	439 (13.7)
Alzheimer disease	1,126 (4.1)	352 (4.8)	207 (3.0)	674 (4.1)	132 (4.3)	320 (4.5)	78 (3.7)	207 (3.5)	163 (5.1)
Vascular dementia	451 (1.6)	159 (2.2)	76 (1.1)	267 (1.6)	49 (1.6)	138 (1.9)	19 (0.9)	85 (1.4)	76 (2.4)

Abbreviations: BMI = body mass index; CVD = cardiovascular disease; MET = metabolic equivalent task.

a Variables are presented as mean ± SD or n (%).

**Table 2:** Baseline Characteristics by Categories of Milk, Fermented Milk, and Butter Consumption^a^

Characteristics	High-fat milk		Low-fat milk		High-fat fermented milk		Low-fat fermented milk		Butter	
	0 g/d	≥500 g/d	0 g/d	≥500 g/d	0 g/d	≥200 g/d	0 g/d	≥200 g/d	0 g/d	≥40 g/d
Participants, n	1,618	1,017	8,664	2,473	16,312	1,667	18,386	1,719	15,894	2,467
Age, y	57.2 ± 7.1	58.6 ± 7.4	58.2 ± 7.6	58.3 ± 7.6	58.5 ± 7.6	58.2 ± 7.7	58.4 ± 7.7	58.0 ± 7.4	58.4 ± 7.6	58.5 ± 7.4
Female	1,023 (63.2)	402 (39.5)	4,838 (55.8)	1,243 (50.3)	9,298 (57.0)	957 (57.4)	10,354 (56.3)	1,034 (60.2)	9,638 (60.6)	1,001 (40.6)
BMI, kg/m^2^	25.8 ± 4.1	25.3 ± 4.0	25.3 ± 3.9	26.6 ± 4.2	25.9 ± 4.0	25.0 ± 3.7	25.7 ± 4.0	25.7 ± 3.6	26.0 ± 4.0	24.9 ± 3.8
Married	1,004 (62.1)	570 (56.1)	5,267 (60.8)	1,613 (65.2)	10,936 (67.0)	977 (58.6)	11,861 (64.5)	1,129 (65.7)	10,891 (68.5)	1,455 (59.0)
Living alone	433 (26.8)	335 (32.9)	2,476 (28.6)	632 (25.6)	3,850 (23.6)	466 (28.0)	4,553 (24.8)	442 (25.7)	3,478 (21.9)	757 (30.7)
University degree	232 (14.3)	102 (10.0)	1,136 (13.1)	297 (12.0)	1,975 (12.1)	359 (21.5)	2,361 (12.8)	350 (20.4)	1,986 (12.5)	287 (11.6)
Smoking status										
Current	473 (29.2)	503 (49.5)	2,868 (33.1)	770 (31.1)	4,661 (28.6)	467 (28.0)	5,724 (31.1)	331 (19.3)	4,027 (25.3)	1,035 (42.0)
Past	584 (36.1)	233 (22.9)	2,846 (32.9)	837 (33.9)	5,666 (34.7)	584 (35.0)	6,103 (33.2)	713 (41.5)	5,649 (35.5)	751 (30.4)
Never	561 (34.7)	281 (27.6)	2,950 (34.1)	866 (35.0)	5,985 (36.7)	616 (37.0)	6,559 (35.7)	675 (39.3)	6,218 (39.1)	681 (27.6)
Zero-consumers of alcohol	116 (7.2)	111 (10.9)	598 (6.9)	256 (10.4)	1,137 (7.0)	116 (7.0)	1,244 (6.8)	98 (5.7)	1,107 (7.0)	135 (5.5)
High leisure-time physical activity (>50 MET hours/week)	256 (15.8)	192 (18.9)	1,414 (16.3)	446 (18.0)	2,551 (15.6)	317 (19.0)	2,916 (15.9)	329 (19.1)	2,553 (16.1)	416 (16.9)
Heredity score for CVD (>0)	852 (52.7)	518 (50.9)	4,446 (51.3)	1,310 (53.0)	8,714 (53.4)	832 (49.9)	9,686 (52.7)	914 (53.2)	8,632 (54.3)	1,159 (47.0)
Chronic conditions at baseline										
Diabetes	88 (5.4)	34 (3.3)	295 (3.4)	138 (5.6)	870 (5.3)	39 (2.3)	755 (4.1)	85 (4.9)	844 (5.3)	73 (3.0)
Hypertension	981 (60.7)	628 (61.8)	5,283 (61.0)	1,610 (65.1)	10,413 (63.9)	941 (56.5)	11,469 (62.4)	1,012 (58.9)	10,142 (63.9)	1,486 (60.3)
CVD	54 (3.3)	20 (2.0)	220 (2.5)	108 (4.4)	591 (3.6)	32 (1.9)	552 (3.0)	79 (4.6)	562 (3.5)	59 (2.4)
Stroke	15 (0.9)	12 (1.2)	86 (1.0)	40 (1.6)	202 (1.2)	13 (0.8)	194 (1.1)	24 (1.4)	171 (1.1)	30 (1.2)
Cancer	103 (6.4)	48 (4.7)	527 (6.1)	162 (6.6)	1,040 (6.4)	101 (6.1)	1,105 (6.0)	112 (6.5)	1,025 (6.5)	140 (5.7)
Lipid-lowering medication	67 (4.1)	10 (1.0)	178 (2.1)	115 (4.7)	649 (4.0)	25 (1.5)	515 (2.8)	75 (4.4)	684 (4.3)	24 (1.0)
Diet quality index	2.2 ± 1.3	1.4 ± 1.1	1.8 ± 1.3	1.8 ± 1.2	1.9 ± 1.3	2.0 ± 1.3	1.8 ± 1.2	2.3 ± 1.4	2.0 ± 1.3	1.5 ± 1.1
Total energy intake, kcal/d	2,058.9 ± 588.4	2,982.4 ± 816.5	2,318.9 ± 709.1	2,541.0 ± 691.1	2,231.0 ± 655.3	2,511.7 ± 669.7	2,315.2 ± 672.2	2,314.8 ± 642.7	2,192.0 ± 613.5	2,938.1 ± 733.0
All-cause dementia (2014)	102 (6.3)	71 (7.0)	584 (6.7)	200 (8.1)	1,158 (7.1)	118 (7.1)	1,300 (7.1)	125 (7.3)	1,162 (7.3)	195 (7.9)
All-cause dementia (2020)	179 (11.1)	108 (10.6)	968 (11.2)	304 (12.3)	1,918 (11.8)	197 (11.8)	2,139 (11.6)	200 (11.6)	1,917 (12.1)	298 (12.1)
Alzheimer disease	60 (3.7)	34 (3.3)	340 (3.9)	113 (4.6)	679 (4.2)	68 (4.1)	736 (4.0)	79 (4.6)	670 (4.2)	112 (4.5)
Vascular dementia	20 (1.2)	17 (1.7)	140 (1.6)	47 (1.9)	286 (1.8)	22 (1.3)	323 (1.8)	29 (1.7)	282 (1.8)	49 (2.0)

Abbreviations: BMI = body mass index; CVD = cardiovascular disease; MET = metabolic equivalent task.

a Variables are presented as mean ± SD or n (%).

### Dairy and All-Cause Dementia

**Figure 1:**
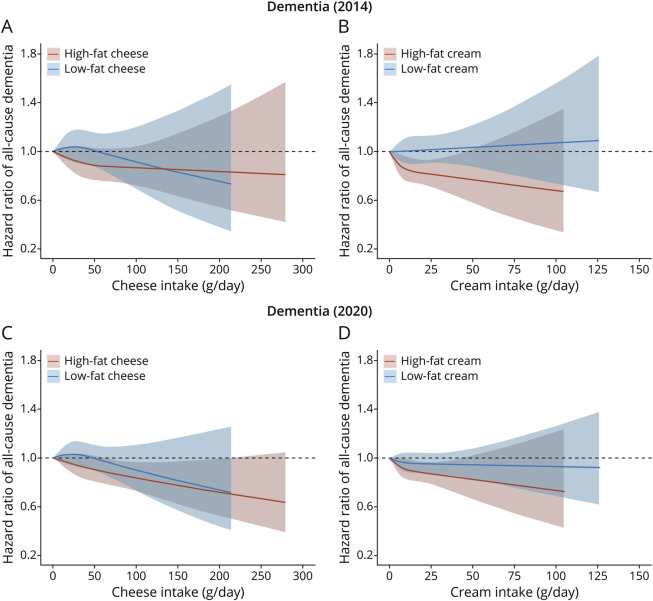
Dose-Response Associations of All-Cause Dementia With High-Fat and Low-Fat Types of Cheese (A and C) and Cream (B and D) The follow-up was censored on December 31, 2014 (A and B). The follow-up was censored on December 31, 2020 (C and D). No consumption was used as reference level (hazard ratio 1). Model 2 covariates were adjusted.

There were no dementia cases at baseline. With follow-up censored on December 31, 2014 (median follow-up of 19.7 years), a total of 1,920 all-cause dementia cases were observed, including 1,126 AD cases and 451 VaD cases (Table 1). When the follow-up period was extended to December 31, 2020 (median follow-up of 24.9 years), we observed 3,208 all-cause dementia cases. In fully adjusted models, inverse associations of all-cause dementia (2020) with high-fat cheese intake (HR per SD increase, 0.95; 95% CI, 0.91–0.99) and high-fat cream intake (HR per SD increase, 0.95; 95% CI, 0.92–0.99) were observed (Table 3). The spline analysis revealed a linear inverse association between high-fat cheese intake and all-cause dementia (2020), whereas a similar but nonsignificant trend was seen for all-cause dementia (2014) (Figure 1). Similar inverse dose-response associations (*p* nonlinear >0.05) were observed between high-fat cream intake and dementia (both 2014 and 2020). Compared with those in the lowest intake group, the HR for all-cause dementia (2020) was 0.87 (95% CI 0.78–0.97, *p*-trend = 0.018) for participants with a high intake of high-fat cheese (≥50 g/d) and 0.84 (95% CI, 0.72–0.98, *p*-trend = 0.052) for participants consuming ≥20 g/d of high-fat cream. Low-fat cheese, low-fat cream, high-fat milk, fermented milk (low-fat and high-fat), and butter showed null associations with all-cause dementia (Table 3, Figure 1, and eFigure 2). Participants consuming ≥500 g/d of low-fat milk (vs no consumption) had a 24% higher risk of all-cause dementia (2014); however, this association was no longer significant for all-cause dementia (2020). Furthermore, when dairy products were analyzed without differentiating fat content, total cheese and cream also showed significant inverse associations (eFigure 3).

**Table 3:** Hazard Ratios (95% CIs) for All-Cause Dementia by Dairy Product Consumption^a^

	N^b^	All-cause dementia (2014)			All-cause dementia (2020)		
		Cases/person-years	Model 1	Model 2	Cases/person-years	Model 1	Model 2
High-fat cheese							
<15 g/d	7,340	609/131,401	Ref	Ref	968/154,210	Ref	Ref
15 to <30 g/d	6,538	483/117,672	0.92 (0.81–1.03)	0.93 (0.82–1.05)	798/139,511	0.93 (0.84–1.02)	0.94 (0.85–1.04)
30 to <50 g/d	6,791	460/124,916	0.92 (0.81–1.04)	0.94 (0.83–1.07)	777/149,554	0.92 (0.83–1.01)	0.93 (0.85–1.03)
≥50 g/d	7,001	368/130,463	0.83 (0.72–0.95)	0.87 (0.75–1.01)	665/157,618	0.84 (0.76–0.94)	0.87 (0.78–0.97)
*p*-trend		—	0.011	0.095	—	0.002	0.018
Per SD increase		—	0.94 (0.89–0.99)	0.96 (0.90–1.02)	—	0.94 (0.90–0.98)	0.95 (0.91–0.99)
Low-fat cheese							
0 g/d	16,318	1,155/293,430	Ref	Ref	1,885/348,319	Ref	Ref
<10 g/d	4,454	282/82,649	0.97 (0.85–1.10)	0.99 (0.86–1.13)	511/99,387	1.05 (0.95–1.15)	1.06 (0.96–1.17)
10 to <30 g/d	3,847	271/71,476	0.99 (0.86–1.13)	1.00 (0.87–1.15)	449/85,414	0.99 (0.89–1.09)	0.99 (0.89–1.10)
≥30 g/d	3,051	212/56,897	1.05 (0.91–1.21)	1.06 (0.91–1.24)	363/67,773	1.06 (0.95–1.19)	1.05 (0.93–1.19)
*p*-trend		—	0.541	0.488	—	0.407	0.560
Per SD increase		—	0.99 (0.94–1.04)	0.99 (0.94–1.04)	—	0.99 (0.96–1.03)	0.99 (0.95–1.03)
High-fat cream							
0 g/d	7,149	549/129,104	Ref	Ref	892/151,362	Ref	Ref
<10 g/d	13,915	955/253,921	0.90 (0.81–1.00)	0.93 (0.84–1.04)	1,580/303,843	0.88 (0.81–0.96)	0.91 (0.84–0.99)
10 to <20 g/d	4,485	291/82,241	0.83 (0.72–0.96)	0.89 (0.76–1.03)	513/98,768	0.87 (0.78–0.97)	0.92 (0.82–1.03)
≥20 g/d	2,121	125/39,186	0.76 (0.62–0.92)	0.82 (0.67–1.00)	223/46,920	0.80 (0.69–0.93)	0.84 (0.72–0.98)
*p*-trend		—	0.003	0.040	—	0.006	0.052
Per SD increase		—	0.91 (0.87–0.96)	0.93 (0.89–0.98)	—	0.94 (0.90–0.98)	0.95 (0.92–0.99)
Low-fat cream							
0 g/d	5,952	370/106,995	Ref	Ref	660/127,580	Ref	Ref
<10 g/d	14,293	933/261,777	0.97 (0.86–1.10)	0.98 (0.86–1.10)	1,579/313,206	0.91 (0.83–0.99)	0.91 (0.83–1.00)
10 to <20 g/d	4,224	333/77,198	0.97 (0.84–1.13)	0.97 (0.83–1.12)	530/91,578	0.88 (0.79–0.99)	0.88 (0.78–0.99)
≥20 g/d	3,201	284/58,482	1.05 (0.90–1.23)	1.04 (0.88–1.22)	439/68,529	0.94 (0.83–1.07)	0.93 (0.82–1.06)
*p*-trend		—	0.364	0.533	—	0.743	0.580
Per SD increase		—	1.01 (0.97–1.06)	1.01 (0.97–1.05)	—	0.99 (0.96–1.02)	0.98 (0.95–1.02)
High-fat milk							
0 g/d	1,618	102/30,178	Ref	Ref	179/35,748	Ref	Ref
<250 g/d	22,839	1,563/417,901	0.99 (0.81–1.21)	0.98 (0.80–1.20)	2,643/499,169	0.95 (0.82–1.11)	0.96 (0.82–1.11)
250 to <500 g/d	2,196	184/38,753	1.19 (0.93–1.52)	1.18 (0.92–1.52)	278/45,506	1.06 (0.88–1.28)	1.04 (0.86–1.27)
≥500 g/d	1,017	71/17,620	1.12 (0.82–1.52)	1.06 (0.77–1.47)	108/20,470	1.02 (0.80–1.30)	0.95 (0.74–1.22)
*p*-trend		—	0.034	0.107	—	0.141	0.552
Per SD increase		—	1.05 (1.00–1.10)	1.04 (0.99–1.10)	—	1.02 (0.99–1.06)	1.01 (0.97–1.05)
Low-fat milk							
0 g/d	8,664	584/155,468	Ref	Ref	968/184,595	Ref	Ref
<250 g/d	10,517	689/195,388	0.96 (0.86–1.08)	1.02 (0.91–1.15)	1,197/234,673	0.98 (0.90–1.07)	1.01 (0.93–1.11)
250 to <500 g/d	6,016	447/110,013	0.96 (0.85–1.09)	1.01 (0.89–1.16)	739/130,465	0.98 (0.89–1.07)	0.98 (0.88–1.09)
≥500 g/d	2,473	200/43,584	1.21 (1.03–1.43)	1.24 (1.04–1.48)	304/51,159	1.15 (1.01–1.30)	1.11 (0.97–1.28)
*p*-trend		—	0.083	0.050	—	0.149	0.368
Per SD increase		—	1.03 (0.98–1.08)	1.04 (0.99–1.09)	—	1.02 (0.99–1.06)	1.01 (0.97–1.05)
High-fat fermented milk							
0 g/d	16,312	1,158/295,061	Ref	Ref	1,918/349,856	Ref	Ref
<100 g/d	6,288	403/116,502	1.01 (0.90–1.13)	1.03 (0.92–1.16)	679/140,297	0.98 (0.90–1.07)	0.99 (0.91–1.09)
100 to <200 g/d	3,403	241/62,492	0.93 (0.81–1.07)	0.98 (0.85–1.13)	414/74,501	0.96 (0.86–1.07)	0.98 (0.88–1.10)
≥200 g/d	1,667	118/30,397	1.00 (0.83–1.21)	1.08 (0.89–1.32)	197/36,239	0.98 (0.85–1.14)	1.02 (0.88–1.18)
*p*-trend		—	0.578	0.675	—	0.541	0.995
Per SD increase		—	0.99 (0.95–1.04)	1.02 (0.97–1.06)	—	0.99 (0.96–1.03)	1.00 (0.97–1.04)
Low-fat fermented milk							
0 g/d	18,386	1,300/330,817	Ref	Ref	2,139/392,108	Ref	Ref
<100 g/d	4,436	260/82,921	0.93 (0.81–1.06)	0.95 (0.83–1.09)	489/99,945	1.01 (0.91–1.11)	1.03 (0.93–1.14)
100 to <200 g/d	3,129	235/58,591	1.02 (0.89–1.17)	1.08 (0.94–1.25)	380/70,430	0.97 (0.87–1.08)	1.01 (0.90–1.13)
≥200 g/d	1,719	125/32,122	1.01 (0.84–1.22)	1.11 (0.92–1.34)	200/38,409	0.96 (0.83–1.11)	1.02 (0.88–1.18)
*p*-trend		—	0.820	0.176	—	0.478	0.804
Per SD increase		—	1.00 (0.96–1.05)	1.03 (0.98–1.08)	—	0.98 (0.95–1.02)	1.00 (0.96–1.03)
Butter							
0 g/d	15,894	1,162/290,436	Ref	Ref	1,917/345,280	Ref	Ref
<20 g/d	6,089	350/112,200	0.84 (0.75–0.95)	0.87 (0.77–0.99)	650/134,856	0.93 (0.85–1.02)	0.96 (0.87–1.05)
20 to <40 g/d	3,220	213/58,153	0.97 (0.84–1.12)	0.99 (0.85–1.14)	343/69,220	0.94 (0.84–1.05)	0.95 (0.84–1.06)
≥40 g/d	2,467	195/43,663	1.16 (0.98–1.36)	1.15 (0.98–1.36)	298/51,537	1.06 (0.93–1.20)	1.04 (0.91–1.19)
*p*-trend		—	0.115	0.120	—	0.678	0.812
Per SD increase		—	1.04 (0.99–1.09)	1.04 (0.99–1.09)	—	1.01 (0.97–1.05)	1.00 (0.97–1.04)

a Model 1: adjusted for age, sex, dietary assessment version (method), season, and total energy intake. Model 2: model 1 plus educational level, leisure-time physical activity, smoking status, alcohol consumption, family history of cardiovascular disease, marriage, living alone, diet quality index, body mass index, hypertension, and mutually adjusted dairy products.

b The number of participants in each category.

### Dairy and Dementia Subtypes

In fully adjusted models, participants with a high intake of high-fat cheese (≥50 g/d) had a 29% lower risk of VaD (HR 0.71, 95% CI 0.52–0.96, *p*-trend = 0.050) compared with those with a lower intake (Table 4). High-fat cream intake was inversely associated with the risk of AD and VaD when modeled as a continuous variable. Compared with those with no butter consumption, high intake (≥40 g/d) was associated with higher AD risk (HR 1.27, 95% CI 1.02–1.58, *p*-trend = 0.012). No other dairy products were associated with AD or VaD risk.

**Table 4:** Hazard Ratios (95% CIs) for Dementia Subtypes (2014) by Dairy Product Consumption^a^

	Categories				*p*-trend	Continuous	
High-fat cheese	<15 g/d	15 to <30 g/d	30 to <50 g/d	≥50 g/d	—	Per SD increase	*p* Value
AD/VaD (n)^b^	352/159	298/106	269/110	207/76			
AD	1.00	0.99 (0.84–1.16)	0.97 (0.81–1.14)	0.87 (0.72–1.06)	0.155	0.96 (0.89–1.04)	0.291
VaD	1.00	0.80 (0.62–1.03)	0.88 (0.68–1.14)	0.71 (0.52–0.96)	0.050	0.89 (0.78–1.01)	0.066
Low-fat cheese	0 g/d	<10 g/d	10 to <30 g/d	≥30 g/d	—	Per SD increase	*p* Value
AD/VaD (n)	674/267	164/67	156/68	132/49			
AD	1.00	0.92 (0.77–1.09)	0.95 (0.79–1.14)	1.09 (0.90–1.33)	0.396	1.02 (0.96–1.09)	0.545
VaD	1.00	1.13 (0.86–1.48)	1.16 (0.88–1.53)	1.12 (0.81–1.54)	0.447	0.97 (0.87–1.08)	0.580
High-fat cream	0 g/d	<10 g/d	10 to <20 g/d	≥20 g/d	—	Per SD increase	*p* Value
AD/VaD (n)	320/138	552/234	176/60	78/19			
AD	1.00	0.88 (0.77–1.02)	0.87 (0.72–1.05)	0.83 (0.64–1.07)	0.166	0.93 (0.87–1.00)	0.036
VaD	1.00	0.99 (0.79–1.23)	0.80 (0.59–1.10)	0.56 (0.34–0.91)	0.008	0.84 (0.74–0.95)	0.005
Low-fat cream	0 g/d	<10 g/d	10 to <20 g/d	≥20 g/d	—	Per SD increase	*p* Value
AD/VaD (n)	207/85	551/219	205/71	163/76			
AD	1.00	0.98 (0.83–1.15)	1.01 (0.83–1.23)	1.02 (0.83–1.27)	0.648	1.00 (0.95–1.06)	0.868
VaD	1.00	1.10 (0.85–1.42)	0.98 (0.71–1.36)	1.26 (0.92–1.74)	0.294	1.07 (0.99–1.15)	0.085
High-fat milk	0 g/d	<250 g/d	250 to <500 g/d	≥500 g/d	—	Per SD increase	*p* Value
AD/VaD (n)	60/20	924/373	108/41	34/17			
AD	1.00	0.99 (0.76–1.29)	1.23 (0.88–1.71)	0.98 (0.63–1.53)	0.275	1.02 (0.95–1.10)	0.621
VaD	1.00	1.20 (0.76–1.89)	1.19 (0.68–2.07)	0.99 (0.50–1.96)	0.609	0.98 (0.88–1.09)	0.703
Low-fat milk	0 g/d	<250 g/d	250 to <500 g/d	≥500 g/d	—	Per SD increase	*p* Value
AD/VaD (n)	340/140	414/160	259/104	113/47			
AD	1.00	0.99 (0.85–1.15)	0.98 (0.82–1.17)	1.26 (1.00–1.59)	0.130	1.01 (0.95–1.09)	0.701
VaD	1.00	1.04 (0.82–1.33)	0.94 (0.71–1.24)	1.02 (0.71–1.46)	0.773	1.01 (0.92–1.12)	0.826
High-fat fermented milk	0 g/d	<100 g/d	100 to <200 g/d	≥200 g/d	—	Per SD increase	*p* Value
AD/VaD (n)	679/286	233/91	146/52	68/22			
AD	1.00	0.98 (0.84–1.14)	0.98 (0.82–1.18)	1.07 (0.82–1.38)	0.797	1.01 (0.95–1.08)	0.725
VaD	1.00	1.01 (0.79–1.29)	0.88 (0.65–1.19)	0.82 (0.53–1.27)	0.253	0.96 (0.86–1.06)	0.410
Low-fat fermented milk	0 g/d	<100 g/d	100 to <200 g/d	≥200 g/d	—	Per SD increase	*p* Value
AD/VaD (n)	736/323	159/53	152/46	79/29			
AD	1.00	0.98 (0.82–1.17)	1.16 (0.97–1.40)	1.18 (0.93–1.50)	0.060	1.06 (1.00–1.12)	0.059
VaD	1.00	0.85 (0.63–1.15)	0.91 (0.66–1.25)	1.10 (0.75–1.63)	0.964	0.98 (0.89–1.09)	0.734
Butter	0 g/d	<20 g/d	20 to <40 g/d	≥40 g/d	—	Per SD increase	*p* Value
AD/VaD (n)	670/282	205/76	139/44	112/49			
AD	1.00	0.86 (0.73–1.01)	1.13 (0.94–1.36)	1.27 (1.02–1.58)	0.012	1.06 (0.99–1.13)	0.074
VaD	1.00	0.82 (0.63–1.06)	0.81 (0.59–1.12)	1.06 (0.76–1.46)	0.941	1.03 (0.94–1.13)	0.545

Abbreviations: AD = Alzheimer disease; VaD = vascular dementia.

a Model 2 covariates were adjusted.

b The number of AD and VaD cases in each category.

### Subgroup and Substitution Analyses

**Figure 2:**
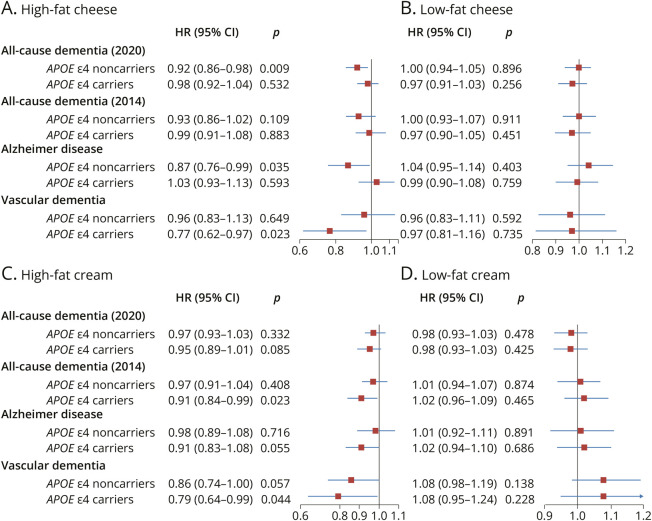
Association Between Per SD Increase of Cheese and Cream Consumption and All-Cause Dementia and Dementia Subtypes, Stratified by *APOE* ε4 Status Model 2 covariates were adjusted. *APOE* ε4 status modified the association between high-fat cheese and Alzheimer disease (*p*-interaction = 0.014). For all other interactions, *p*-interaction >0.05. HR = hazard ratio.

An inverse association between high-fat cheese and AD was found among *APOE* ε4 noncarriers (HR 0.87, 95% CI 0.76–0.99, *p*-interaction = 0.014) (Figure 2). The associations of high-fat and low-fat cheese and cream consumption with all-cause dementia were not modified by sex, age, educational level, or diet quality index (eTable 1). Higher intake of high-fat fermented milk showed a protective association with all-cause dementia among those with higher education levels (*p*-interaction = 0.003). A similar protective association was found for higher butter intake and all-cause dementia among participants with better diet quality (*p*-interaction = 0.003) (eTable 2).

When replacing 20 g/d of high-fat cheese intake with the equivalent amount of other foods, increased all-cause dementia risk was observed for milk, fermented milk, high-fat red meat, and processed meat (eFigure 4). Increased all-cause dementia risk was observed for more foods when replacing high-fat cream.

The results remained robust after excluding cases with prevalent CVD, cancer, or diabetes, and after further adjustment for multiple chronic conditions (eTable 3). Notably, stronger inverse associations between high-fat cheese intake and all-cause dementia (both 2014 and 2020) were observed after excluding dementia cases that occurred within the first 10 years of follow-up. Restricting the analysis to those who reported no apparent change in their diet during the 5-year follow-up examination resulted in weakened associations between high-fat cheese intake and all-cause dementia (2020). When modeled as categories, the observed associations with all-cause dementia (2020) (HR 0.89, 95% CI 0.77–1.02, *p*-trend = 0.090) and VaD (HR 0.69, 95% CI 0.48–1.01; *p*-trend = 0.050) in the highest intake group (≥50 g/d) weakened and became nonsignificant.

## Discussion

In this Swedish population, we found that higher intake of high-fat cheese and high-fat cream was associated with a lower risk of all-cause dementia, independent of lifestyle factors, other dairy products, and diet quality. High-fat cheese intake was associated with a lower risk of AD among *APOE* ε4 noncarriers. The intake of other dairy products was not associated with all-cause dementia risk.

Our results are in line with findings from a Finnish cohort study,^15^ which showed a lower risk of dementia with higher cheese intake (quartile 4 vs quartile 1: HR 0.72, 95% CI 0.52–0.99), and a UK Biobank–based study,^19^ which observed a lower risk with higher intake frequency (once a week vs no consumption: HR 0.81, 95% CI 0.67–0.98). By contrast, another Japanese cohort study,^16^ with 5 years of follow-up and 946 incident dementia cases, did not find a significant association, possibly because of limited statistical power and potential reverse causation. Furthermore, 4 cross-sectional studies from Japan, the Netherlands, or the United Kingdom consistently supported that higher cheese intake was associated with better cognitive function in older adults.^4,20-22^ Our study strengthens and extends previous evidence with the longest follow-up period to date and a large number of dementia cases, suggesting that the observed inverse association was mainly driven by cheese with higher fat content. In the dose-response relationship, a lower risk of all-cause dementia was observed when daily intake of high-fat cheese exceeded 50 g/d, which is the 75th percentile in this population and not a rare, difficult-to-meet amount.

In the MIND diet, cheese is categorized as an unhealthy food to limit, mainly because of concerns about its high amount of saturated fat.^18,40^ However, subsequent new evidence from RCTs consistently suggested that regular-fat cheese did not cause adverse changes in blood lipid profiles compared with low-fat cheese or a control diet.^41-43^ Indeed, regular-fat cheese was even found to demonstrate the greatest metabolic health benefits in animal models, including increased fecal fat and energy excretion, as well as beneficial changes in gut microbiota, compared with reduced-fat cheese and butter.^44^ In Mendelian randomization studies,^45,46^ cheese has been causally associated with a lower risk of diabetes and hypertension, both of which are risk factors of dementia.^47^ We speculate that differences in fat content, other nutrients (e.g., vitamin K2), and the food matrix between high-fat and low-fat cheese have the potential to explain our observed protective association.

It is possible that higher high-fat cheese intake served as a surrogate marker for a healthier diet, lifestyle, better health condition, or other unmeasured protective confounding factors. Our data showed that individuals with higher high-fat cheese intake were more likely to be current or past smokers, with higher alcohol consumption and lower diet quality. Consumers of higher amounts of high-fat cheese also tended to be younger, with lower BMI; higher education levels; a lower prevalence of diabetes, hypertension, and CVD; and less use of lipid-lowering medication. However, similar results were observed after adjusting for these risk factors or mediators. In addition, the distribution of these factors was not unique to high-fat cheese; those with higher intake of other high-fat dairy (e.g., high-fat fermented milk) exhibited a similar distribution pattern of these factors. Therefore, the likelihood of protective confounding factors that meet the condition—being specific to high-fat cheese intake but unrelated to the intake of other high-fat foods—is low.

Reverse causation has always been a concern in studies exploring risk factors of dementia, considering its long preclinical phase. In sensitivity analyses, excluding incident dementia cases within the first 10 years of follow-up resulted in stronger inverse associations between high-fat cheese intake and dementia in both 2014 and 2020 data sets. Cognitive decline during the preclinical phase of dementia may affect the accurate reporting or changes in dietary habits. The stronger association after aforementioned exclusions further underscores the necessity of long follow-up periods. Our assessment of low-fat dairy intake may not represent its long-term intake because some participants may have switched from high-fat to low-fat types after a disease diagnosis. However, excluding prevalent cases of CVD, cancer, or diabetes at baseline yielded similar results.

We observed that high-fat cheese intake was linked to a lower AD risk in *APOE* ε4 noncarriers. Although previous studies suggest that the *APOE* genotype may modify the association between diet and dementia or AD, the variability in observed findings limits the ability to draw firm conclusions. Higher intake of fatty fish and fish, or adherence to a healthy dietary pattern, has been associated with a lower risk of all-cause dementia or AD among *APOE* ε4 noncarriers.^23-25^ By contrast, adverse associations between dementia risk and a refined carbohydrate-rich diet, or a Western dietary pattern, have been reported among *APOE* ε4 carriers.^25,26^ However, no interaction with *APOE* ε4 status was observed for cheese.^15^ Differences in sample size (most studies had fewer than 400 all-cause dementia cases), participant age, diet pattern, and types of food may explain these discrepancies.

Our study contributes novel cohort evidence on the association between cream consumption and dementia. Our observed inverse association between high-fat cream and all-cause dementia requires caution in interpretation and further replications because the association found with dementia (2014) seemed to weaken when follow-up was extended to 2020 (despite an increase in statistical power). Besides, in the validation study assessing reproducibility, cream showed lower correlation coefficients (female 0.42, male 0.48) compared with cheese (0.71), indicating a relatively higher measurement error and a limited capacity of our dietary assessment method to capture its long-term intake.

Milk and fermented milk were not associated with either all-cause dementia or dementia subtypes. The results of a UK Biobank–based cohort study are consistent with our finding, which observed null associations between skimmed milk and full-cream milk and the risk of all-cause dementia, AD, and VaD.^17^ Similar findings were observed for milk in 2 cohort studies.^15,16^ The only study on yogurt suggested a null association with dementia risk.^16^ A recent review also noted that milk is either unrelated to cognitive function or potentially harmful, while the findings for yogurt remain inconsistent.^14^ Our finding of a protective association between high-fat fermented milk and dementia among individuals with higher education levels may be a chance finding and warrants further investigation.

We found that butter intake was positively associated with AD risk, but among individuals with high diet quality, it was inversely associated with the risk of all-cause dementia. Butter is a high-fat dairy product, containing approximately 80% fat. In our study, participants with higher diet quality tended to consume less fat. Given the reported U-shaped association between fat intake and dementia risk,^48^ butter consumption might be protective when included in a low-fat diet, whereas in a diet already high in fat, it may increase the risk. Epidemiologic evidence linking butter to dementia is currently lacking, and this interpretation remains speculative. Further research is needed to confirm these observations. Still, these findings underscore the importance of considering the overall dietary context when evaluating butter's potential role in dementia risk.

We observed an increased risk of all-cause dementia when high-fat cheese and cream were replaced with most other foods with various fat contents. These findings suggested that focusing on the fat sourced from certain foods, rather than total dietary fat, is more meaningful; this aligns with the previous insight that the specific food sources of dietary fat are more important than its quantity in influencing chronic disease risk.^49^

Key strengths of the study include the validation of register-based dementia diagnosis, population-based prospective design, a long follow-up of up to 30 years, a low proportion of loss to follow-up (0.75%), a broad intake range (with the upper limit of high-fat cheese close to 280 g/d), and the use of a 7-day food diary.

Several limitations warrant consideration. First, owing to the observational nature of the study, potential residual or unmeasured confounding precludes the determination of causation. We calculated the E-value to examine the potential impact of unmeasured confounders.^50^ The E-value was 1.56 for the observed HR of 0.87 for consuming 50 g/d or more of high-fat cheese and all-cause dementia, meaning that an unmeasured confounder would need to have an HR of at least 1.56 to overturn this association toward null; this value is higher than the HR of several well-established risk factors, including current smoking (1.14), hypertension (1.15), and diabetes (1.43). Second, diet was assessed only once at baseline, and changes in consumption may occur over the follow-up period. However, restricting the study sample to participants with no substantial diet change at the 5-year follow-up examination yielded weakened point estimates and altered statistical significance. Third, more detailed information on cheese and cream intake, such as fat content when exceeding 20% (for finer classification), specific types, and ways of consuming, was not collected in this study. Fourth, the baseline cognitive status of participants, which may influence dietary behaviors and is also a predictor of dementia, was not assessed. Fifth, dementia cases who did not seek diagnosis may have been missed, as all cases were identified through registry records. Finally, because this study only included the Swedish population, caution is needed when generalizing these findings to other populations.

In conclusion, we found that higher intake of high-fat cheese and high-fat cream, but not other dairy products, was associated with a lower risk of all-cause dementia. High-fat cheese intake was associated with a lower risk of AD in *APOE* ε4 noncarriers. Further confirmation of these findings in diverse populations is warranted.

## Glossary

AD: Alzheimer disease
BMI: body mass index
CVD: cardiovascular disease
FFQ: food frequency questionnaire
HR: hazard ratio
ICD: International Classification of Diseases
MDC: Malmö Diet and Cancer
MET: metabolic equivalent task
MIND: Mediterranean Dietary Approaches to Stop Hypertension Intervention for Neurodegenerative Delay
NPR: National Patient Register
RCT: randomized controlled trial
VaD: vascular dementia
